# Invader abundance and contraction of niche breadth during replacement of a native gammarid amphipod

**DOI:** 10.1002/ece3.8500

**Published:** 2022-03-07

**Authors:** W. Ian Montgomery, Robert W. Elwood, Jaimie T. A. Dick

**Affiliations:** ^1^ School of Biological Sciences Institute for Global Food Security Queen's University Belfast Belfast UK

**Keywords:** freshwater gammarids, interspecific competition, invasive species, niche breadth

## Abstract

The introduction of non‐native species to new locations is a growing global phenomenon with major negative effects on native species and biodiversity. Such introductions potentially bring competitors into contact leading to partial or total species replacements. This creates an opportunity to study novel species interactions as they occur, with the potential to address the strength of inter‐ and intraspecific interactions, most notably competition. Such potential has often not been realized, however, due to the difficulties inherent in detecting rapid and spatially expansive species interactions under natural field conditions. The invasive amphipod crustacean *Gammarus pulex* has replaced a native species, *Gammarus duebeni celticus*, in river and lake systems across Europe. This replacement process is at least partially driven by differential parasitism, cannibalism, and intraguild predation, but the role of interspecific competition has yet to be resolved. Here, we examine how abundance of an invasive species may affect spatial niche breadth of a native congeneric species. We base our analyses of niche breadth on ordination and factor analysis of biological community and physical parameters, respectively, constituting a summative, multidimensional approach to niche breadth along environmental gradients. Results derived from biological and environmental niche criteria were consistent, although interspecific effects were stronger using the biological niche approach. We show that the niche breadth of the native species is constrained as abundance of the invader increases, but the converse effect does not occur. We conclude that the interaction between invasive *G*. *pulex* and native *G*. *d*. *celticus* under natural conditions is consistent with strong interspecific competition whereby a native, weaker competitor is replaced by a superior invasive competitor. This study indicates a strong role of interspecific competition, alongside other known interactions such as differential intraguild predation, in rapid and expansive species replacements following biological invasions.

## Graphical abstract

Niche shift may occur generally at an early stage in species replacements of native by an invasive species, as a result of a competitive processes leading to the eventual demise of a weaker competitor faced with a novel, invasive species.

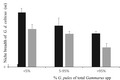

## 1 INTRODUCTION

The introduction of species to new locations is a global phenomenon that often brings potential competitors, predators, and prey into contact (Bengtsson, 1989; Diamond & Case, 1986; Elton, 1958; Gallardo et al., 2016; Sakai et al., 2011; Simberloff et al., 2013). The rate of arrival of invasive alien species is not declining (Hanno Seebens et al., 2017; Seebens et al., 2021), with many horizon scans predicting multiple, new, damaging invasions (Lucy et al., 2020). Thus, we require better understanding and predictions of their effects (Dick et al., 2017). Such redistribution of species may create unplanned, serendipitous field experiments, but these lack the rigorous control and timescale available in the laboratory. Nevertheless, natural and anthropogenic relocations of species have the potential to address questions about the role of interspecific competition in species replacements and in structuring biological communities. This potential has not been realized because of the difficulties inherent in detecting species interactions, particularly interspecific competition, under field conditions (Connell, 1983; Schoener, 1983; Underwood, 1986). Where community data are sufficiently replicated, however, it may be possible to examine intra‐ and interspecific density‐dependent shifts in niche dimensions (Bonesi et al., 2004; Gaston & Spicer, 2001; Jackson et al., 2011; Rosenzweig, 1981; Rosenzweig & Abramsky, 1985; Tran et al., 2015). Using field data, we present evidence, based on the relationships between spatial niche breadth and the abundance of conspecifics and congenerics, that the introduced amphipod, *Gammarus pulex*, is a superior competitor over the native, *G*. *duebeni celticus*, which is being actively replaced by the invader.

The niche concept embraces two principal approaches: the spatial (habitat) niche became closely allied to the Hutchinsonian niche, based on n‐dimensional hyperspace (Hutchinson, 1957), while the Eltonian niche focusses on functional or trophic aspects (Elton, 1927). In recent times, the latter has become much more tractable using stable isotopes (Jackson et al., 2011) and thus there has been an upsurge in interest particularly with regard to the relationships between trophic niche breadth and abundance and range, and predicting success and impacts of invasive species. Prati et al. (2021) used more conventional methods to track changes in diet of Arctic char in response to increased abundance of brown trout. There has been less attention as to how spatial niche breadth might be affected by abundance of conspecifics and potential competitors. In addition, the role of interspecific competition may be masked by focus on other interactions, such as intraguild predation and parasitism (Dick, 2008; Dick et al., 1993; MacNeil, Dick, et al., 2004; MacNeil, Prenter, et al., 2004).

In Ireland, and elsewhere in Europe, the introduced amphipod *Gammarus pulex* has replaced the native, ecologically equivalent *G*. *duebeni celticus* in many freshwater systems (Dick et al., 1994; Stock & Pinkster, 1970; Strange & Glass, 1979). Until 1959, *G*. *pulex* was absent from Ireland, whereas *G*. *d*. *celticus* was common throughout most Irish river systems, including the R. Lagan (Stock & Pinkster, 1970; Strange & Glass, 1979). Sometime, probably in the 1960s, deliberate introductions from North Yorkshire by anglers and fisheries scientists initiated an invasion of the Lagan by *G*. *pulex*. By 1976, *G*. *pulex* was dominant in the lower Lagan and has remained so (Strange & Glass, 1979). This replacement process is associated with parasitism, cannibalism, and intraguild predation and leads to fundamental changes in freshwater invertebrate communities (Dick et al., 1993; Dunn & Dick, 1998; Kelly et al., 2003, 2006; Kelly & Dick, 2005; MacNeil et al., 2003), but the role of interspecific competition has remained elusive (Dick, 2008). This case study thus provides an excellent opportunity to examine the role of species interactions in species replacements on both micro‐ and macro‐geographical scales. Here, we present an extensive field investigation addressing: (1) the relationship between variation in environmental conditions and the abundances of *G*. *pulex* and *G*. *d*. *celticus*; and (2) the interaction of niche breadth and population density of the two species, which yields insights into the process of species replacement and the potential role of interspecific competition. The impact of invasive alien species (IAS) on niche dimensions of direct competitors may also provide insights into processes leading to catastrophic, cascade effects in the wider community and explain the prominence of IAS in the global loss of biodiversity involving diverse habitats and ecosystems (Bellard et al., 2016; Brook et al., 2008; Crooks, 2002; Diamond & Case, 1986; Dickey et al., 2020; Elton, 1958; Estes et al., 2011; Gallardo et al., 2016; Rosenzweig, 2001).

We predict that spatial variation in the abundances of these ecologically similar *Gammarus* spp., being generalist omnivores and predators (MacNeil et al., 1997), is not associated strongly with immediate environmental conditions within natural freshwater systems. This is expected especially where samples are taken from similar locations, rather than from the whole spectrum of conditions within a catchment. We predict that, where replacement of one species by an introduced species is occurring, the niche breadth of the species that is being replaced shrinks as the population density of the invading species increases. Conversely, niche breadth of the invasive species is unaffected by population abundance of its congener. If species replacement is due to some factor other than interspecific competition, niche breadth of the species with the decreasing range should not be affected by the abundance of the species with the expanding range, or the effects of both species should be similar and cumulative. Thus, we expect the weaker competitor and the species that is apparently being replaced in the field, *G*. *d*. *celticus*, to have niche breadth that is influenced by abundance of *G*. *pulex*. The converse relationship, the impact of *G*. *d*. *celticus* population abundance on niche breadth of *G*. *pulex*, is predicted to be weak or non‐existent.

## 2 METHODS

### 2.1 Study area and field methods

**FIGURE 1:**
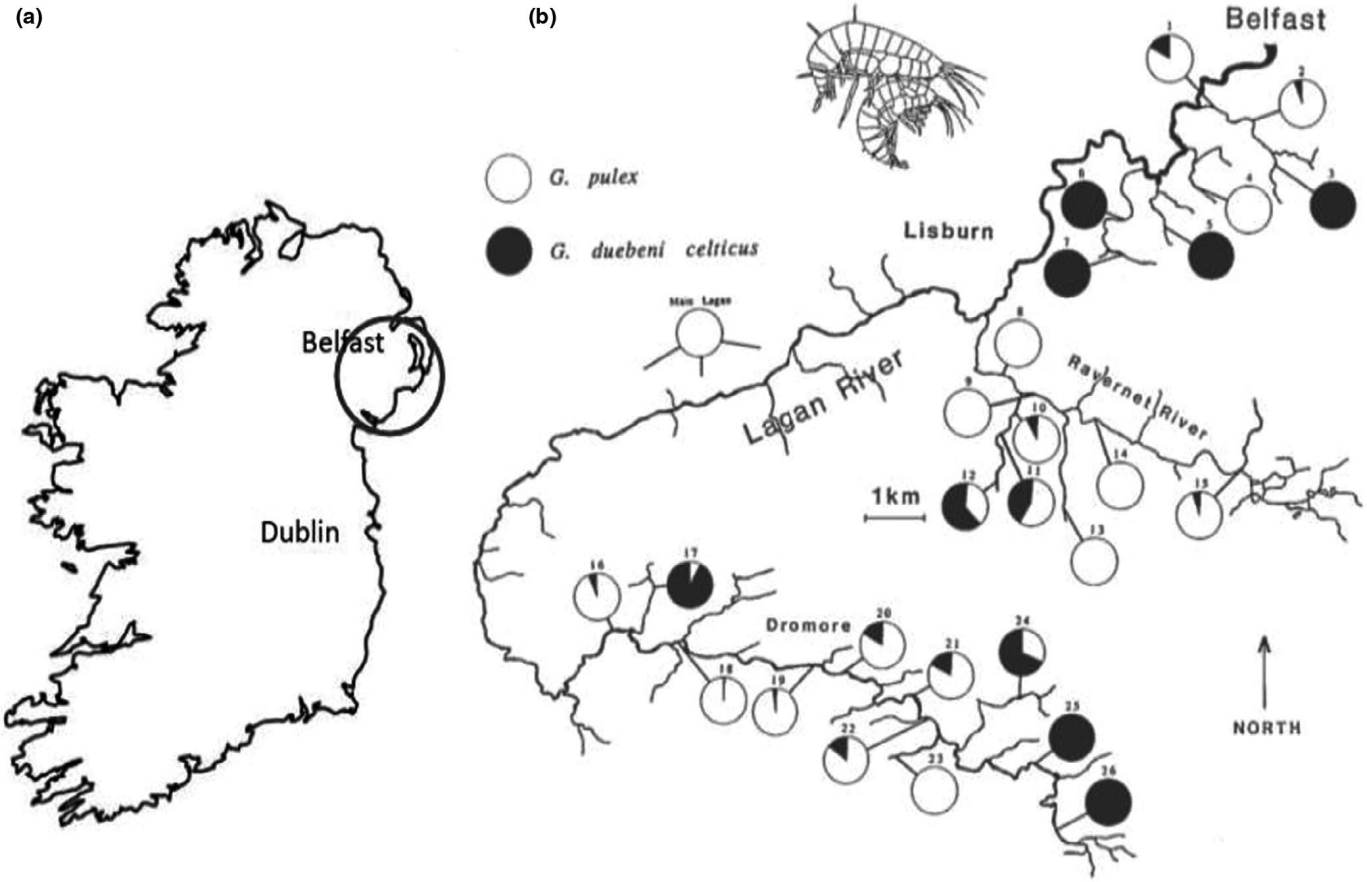
(a) Location (circled) of the study area on the river Lagan in Ireland. (b) The distributions and relative abundances of *Gammarus pulex* and *G*. *duebeni celticus* at 26 sites in the River Lagan in 1993. At each site, 30 × 1 min kick samples were taken at 5‐m intervals working upstream

Abundance of *Gammarus* spp. was examined with respect to variation in environmental conditions and biological community composition in tributaries of the River Lagan, Northern Ireland, between January and April 1993 (Figure 1). A kick sample survey of invertebrates in 1988 suggested that *G*. *d*. *celticus* was able to hold its ground in the upper reaches of Lagan tributaries (Dick et al., 1990). However, a re‐survey in 1991 showed *G*. *pulex* was advancing into upper tributaries, eliminating and replacing *G*. *d*. *celticus* (Dick et al., 1994). These historical surveys were used to identify 26 sites that provided examples of single and mixed species communities of the two species. These sites were predominantly in pastoral farmland on narrow (width <5 m), shallow, non‐tidal tributaries of the main river, suitable for kick sampling. The 1993 survey was fully independent of earlier surveys designed to establish distribution of gammarid species. The later survey was designed to study variation in gammarid abundance and niche breadth.

At each site, 30 invertebrate samples were taken every 5 m moving upstream, comprising a standard kick sample across 1 m of substrate for 1 min by a single, experienced operative using a long‐handled net (20 cm wide) assisted by a second researcher dealing with samples bankside. These samples were fixed on site in a 2% glutaraldehyde solution to avoid any losses due to predation which is frequently observed in live samples (J. Dick, *pers comm*.). Each of the 780 samples was sorted in the laboratory. Gammarids were identified to species and counted. Other invertebrates were identified to family level and counted as an estimate of abundance. Kick sampling is a widely used method for sampling invertebrates in shallow rivers. It provides comparable data on variation in riverine invertebrate communities and abundance to more quantitative but time‐consuming methods (Brua et al., 2011; Everall et al., 2017; Funnell et al., 2020).

Environmental variables were measured or scored at each of the 30 sample points at each site (Table S1). The survey revealed a high degree of heterogeneity with respect to both biotic and physical environmental variables. Depth and speed change rapidly in tributaries in response to rainfall. Thus, on completion of the main survey, each site was resurveyed (10 replicates per site) to establish baselines for these parameters over three days with no rain and these data were used to account for temporal variation in the original measures (Table S1). Sites (*n* = 26) were aggregated on three tributaries: the Minnowburn (7 sites), the Ravernet (8 sites), and Dromore (11 sites; Figure 1). Site and zone were included as random factors in initial analyses. Variation in environmental variables and taxon abundance between sites was examined using independent samples, one‐way ANOVA.

### 2.2 Niche breadth

In order to evaluate spatial niche breadth, it is necessary to measure occurrence along one or more continuous dimensions. Environmental variation at 780 sampling points was assessed using detrended correspondence analysis (referred to as DCA) of data for all invertebrate taxa excluding gammarids (Hill, 1979), and factor analysis (principal components analysis with varimax rotation, referred to as FA) (Bartholomew et al., 2008) of environmental data, that is, all biotic and physical variables (Table S1). DCA has been shown to be a highly reliable and useful tool for data exploration and summary in community ecology (Shaw, 2003). The major strength of this method is that species are arranged along axes representing interpretable environmental gradients. The main problem with DCA is that there is no significance test, but this can be addressed by examining scores on axes to environmental variables using, for example, multiple linear regression. DCA produces a score for each sample on each of four axes (I–IV). Data for *Gammarus* spp. were omitted from DCA such that the axes scores for all sampling points were fully independent of the abundance of these species. FA seeks the least number of factors that can account for the common variance (correlation) of a set of variables and to identify complex interrelationships among items and group items that are part of unified concepts (Polit & Beck, 2012). The researcher makes no *a priori* assumptions about relationships among factors, for example, number of significant factors. The Kaiser criterion of removing components with eigenvalues under 1.0 is often the default in statistical software. It is used here to facilitate selection of factors and the calculation of niche breadth incorporating as much variation in the dataset as possible. It is not recommended when used as the sole cutoff criterion for estimating the number of factors, as it tends to over‐extract factors (Banderloeros & Boehm‐Kaufman, 2008). Gammarid abundance at the site level was estimated as total number in the 30 samples at each site. Stepwise, forward selection using *F* values (entry *p* < .05 and removal *p* < .1), multiple regression analyses were used to estimate the strength of relationship of log _10_‐transformed numbers of *G*. *d*. *celticus* or *G*. *pulex* and scores on axes I‐IV from DCA and factors 1–5 from FA, that is, the nature and extent of the relationship between gammarid abundance and environmental heterogeneity. Regression coefficients (b), their standard errors, *t* for tests of b vs 0, cumulative *r* ^2^ × 100 (=variance accounted for by regression), and variance ratio *F* for overall significance of regression models are presented for each of four analyses. DCA and FA provide an insight into the influence of environment and species distribution and abundance. We use these methods to investigate abundance effects of one gammarid species on another's niche breadth.

Doledec et al. (2000) critically review the history of attempts to quantify the Hutchinsonian niche parameters on multiple dimensions (Hutchinson, 1957). No single method is supported and Doledec et al. (2000) state that “there is a need for a method that addresses the various responses of the species to the environment and at the same time gives a fair vote to all sampling units.” Their method elegantly extends earlier measures to species assemblages with a focus on distance between marginal occurrence and mean of all samples. Here, the interest is just two species and the environmental range is limited. Thus, we use a simple method that assumes species are organized on gradients that are orthogonal but the species distributions are neither random nor normal. To quantify niche breadth, scores on each axis (DCA) or factors in FA were divided into 30 to 45 equal resource states. The number of *Gammarus* occurring in each resource state, which indicates resource utilization and, hence, niche breadth, was calculated along each axis (DCA) and factor (FA) for each site. Niche breadth of *G*. *pulex* and *G*. *d*. *celticus* was calculated for each site, where one or both species occurred (25 of the 26 sites; see below), using Nei and Roychoudhury (1974) unbiased estimate of the Simpson–Yule index. This was inverted (Rosenzweig & Abramsky, 1985) to provide a meaningful interpretation as “the number of equally common categories.” Scores on axes and factors are orthogonal and, hence, represent independent measures of resource utilization. Thus, overall niche breadth of each species was obtained by summing niche breadths across the axes from DCA and, separately, factors from FA. A weighted overall niche breadth was also computed using the eigenvalues, which indicate the relative importance of dimensions in both DCA and FA. Data analyses were conducted using IBM Corp (2017), StatSoft Inc (1991), and Ter Braak (1991). The threshold of significance was *p* = .05.

## 3 RESULTS

### 3.1 Site effects

Two gammarid species, *Gammarus pulex* and *G*. *d*. *celticus*, and 42 other families of freshwater invertebrates were recorded across 780 sample points at 26 sites. Abundance of both species of *Gammarus* differed significantly among sites in univariate ANOVA (Table S2). The percentage of *G*. *pulex* in *Gammarus* spp as a whole increased significantly at 7 of the 26 sites surveyed between 1988–1989 and 1993 and decreased at only one site Dick et al. (1994). All but 6 of the 42 other taxa (Ptychopteridae, Syrphidae, Trichoceridae, Helodidae, Coenagriidae, and Oribatei) differed significantly in mean abundance among sites. All continuous environmental variables also differed significantly among the 26 sites (Table S3). Substrate conditions, that is, dominant substrate and number of substrates, also varied markedly among sites. The present survey, although restricted to the non‐tidal tributaries of the Lagan system, suggests a high degree of heterogeneity with respect to both the invertebrate community and biotic and physical environment, and the ongoing invasion by *G*. *pulex* of the Lagan system.

### 3.2 Abundance relationships in biota

There was a single *G*. *d*. *celticus* and no *G*. *pulex* at site 6 (Figure 1b); this site, therefore, was eliminated from the analyses of niche breadth. There was also a single *G*. *d*. *celticus* at site 4 but this site yielded 2228 *G*. *pulex* and was thus treated as a monospecific *G*. *pulex* site. Thus, the survey produced data for 15 mixed *Gammarus* species, 5 *G*. *pulex*, and 5 *G*. *d*. *celticus* sites (Figure 1b). Abundances of *G*. *pulex* and *G*. *d*. *celticus* were negatively correlated, but not significantly (Spearman rank correlation *r_s_* = −0.367, *p* = .071). *G*. *pulex* was numerous when present, such that its overall abundance in shared sites was highly variable but similar to those where replacement was complete, (mean for sites with between 5% and 95% *G*. *pulex*, 781.25 SE 226.11, and sites with more 95% *G*. *pulex*, mean 764.67 SE 255.92, respectively; Mann–Whitney *U* = 96; *p* = .11). The presence of *G*. *pulex* was associated with lower abundance of *G*. *d*. *celticus* such that sites dominated by *G*. *pulex* (more than 95% of all gammarids) had fewer *G*. *d*. *celticus* than sites with <5% *G*. *pulex* (mean 3.13 SE 1.62, and 559.8 SE 238.01, respectively; Mann–Whitney *U* = 2, *p* < .001). Thus, the populations of *G*. *pulex* and *G*. *d*. *celticus* tended to be biased toward numerical superiority (more than 90%) of one or other species, with only 6 sites having a ratio of *G*. *pulex* to *G*. *d*. *celticus* between 9:1 and 1:9 (Figure 1b).

### 3.3 Gammarid abundance, ordination, and environmental conditions

Initial analyses were conducted using spatial parameters “site” (*n* = 26) and “zone” (*n* = 3) as random factors (see *Methods*). Zone showed no effect in these analyses and was dropped. Variation in abundance of *G*. *pulex* and *G*. *d*. *celticus* was weakly although significantly related to scores on ordination axes 1 to 4 (Table 1a) and Factors 1 to 5 (Table 1b). The latter relationships were stronger than the former (c.f. Table 1a with 1b). However, variation in the environmental variables measured (Table S1) explained <15% and 11% of the variation in abundance of *G*. *pulex* and *G*. *d*. *celticus*, respectively (Table 1).

**TABLE 1:** Results of multiple regression analyses of abundance of *Gammarus pulex* and *G*. *duebeni celticus* on measurements of variation in environmental heterogeneity in the River Lagan system (see Figure 1). *F* is a test of the significance of final model and cumulative r‐squared is an estimate in the variation in abundance accounted for by the terms (axes or factors) as added to the model

(a) Independent variables; scores on axes 1 to 4 from DCA					
	Coef. (b)	SE	*t*	*p*	Cum *r* ^2^ × 100
Dependent *G*. *pulex*		*F* = 32.30, df 2,777, *p* < .0001			
Constant	0.815				
Axis 1	−2.88	0.0004	−7.514	<.0001	5.037
Axis 2	1.72	0.0004	4.713	<.0001	7.676
Dependent *G*. *d*. *celticus*		*F* = 26.58, df 2,777, *p* < .0001			
Constant	0.626				
Axis 4	−1.63	0.003	−4.984	<.0001	3.538
Axis 1	1.43	0.0003	4.875	<.0001	6.402

(b) Independent variables; scores on factors 1 to 5 from FA					
	Coef. (b)	SE	*t*	*p*	Cum *r* ^2^ × 100
Dependent *G*. *pulex*		*F* = 29.87, df 3,776, *p* < .0001			
Constant	0.812				
Factor 1	−0.17	0.024	−7.091	<.0001	5.810
Factor 5	0.14	0.024	5.945	<.0001	9.893
Factor 3	0.05	0.024	1.992	<.05	10.35
Dependent *G*. *d*. *celticus*		*F* = 33.12, df 4,775, *p* < .0001			
Constant	0.464				
Factor 3	0.13	0.018	6.934	<.0001	5.299
Factor 4	−0.12	0.018	−6.465	<.0001	9.905
Factor 1	−0.10	0.018	−5.323	<.0001	13.026
Factor 2	−0.07	0.018	−3.775	<.0002	14.597

Factor normalized loadings indicate their relationship to measured environmental variables (Table 2) and the relationships between factors and axes are further examined in Table 3. These relationships may be positive or negative (Tables 2 and 3). Negative relationships (−ve) are indicated in the text. All others were positive. Factors 1 to 5 were related to (1) substrate heterogeneity and dominance, (2) live vegetation and leaf litter, (3) stream width and depth, (4) grassy versus woody banks, and (5) twig litter. Axis 1 was influenced most strongly by width, depth, and grassy banks, factors 3 and 4 (−ve); axis 2 by substrate (−ve) and grass vs woody cover (−ve); axis 3 by substrate and width and depth (−ve); and axis 4 by substrate (−ve) and twig litter (Table 3).

**TABLE 2:** Eigenvalues and normalized factor loadings (−1 to 1) for factors 1 to 5 from FA indicating the relationship between environmental data at freshwater sampling sites on the River Lagan and factors 1 to 5 (eigenvalues >1). Values in bold indicate loadings >−0.5 or <−0.5

Eigenvalues	2.097	1.816	1.373	1.298	1.039
Environmental variables	Factor 1	Factor 2	Factor 3	Factor 4	Factor 5
Width	−0.01	0.09	**−0.88**	0.04	0.10
Substrate heterogeneity	**−0.85**	−0.07	0.08	0.06	−0.10
Substrate grade dominance	**−0.88**	0.02	−0.03	−0.13	−0.08
Live vegetation	−0.14	0**.85**	−0.08	0.11	−0.19
Grass cover	−0.01	0	0.25	0**.77**	0.11
Bush cover	0.04	−0.03	0.25	**−0.63**	−0.09
Tree cover	−0.03	−0.11	0.15	**−0.62**	0.3
Leaf litter	0.05	0**.88**	**0**.04	0	0.08
Twig litter	−0.05	0.06	0.05	−0.01	**−0.93**
Depth	0.21	−0.06	**−0.84**	**0**.09	−0.07
Speed	**−0.52**	0.20	0.20	0.13	0.18

**TABLE 3:** Correlation (Spearman's rank) between sample scores based on DCA of invertebrate community data (axes) and factor scores (FA) from analysis of biotic and abiotic environmental variables. Factors 1 to 5 interpretation based on normalized loadings (Table 2)

Axis	Eigenvalue	Factor	Interpretation	*r_s_*	*p*<
1	0.483	1	Substrate heterogeneity, dominance	0.015	
		2	Live vegetation and leaf litter	0.037	
		3	Width and depth	0.240	.001
		4	Grass versus woody cover	−0.220	.001
		5	Twig litter	−0.081	.05
2	0.254	1	Substrate heterogeneity, dominance	−0.299	.001
		2	Live vegetation and leaf litter	−0.055	.001
		3	Width and depth	0.127	.001
		4	Grass versus woody cover	−0.288	.001
		5	Twig litter	0.172	.001
3	0.117	1	Substrate heterogeneity, dominance	0.347	.001
		2	Live vegetation and leaf litter	−0.156	.001
		3	Width and depth	0.268	.001
		4	Grass versus woody cover	0.068	
		5	Twig litter	−0.187	.001
4	0.063	1	Substrate heterogeneity, dominance	−0.189	.001
		2	Live vegetation and leaf litter	0.070	
		3	Width and depth	0.010	
		4	Grass versus woody cover	−0.045	
		5	Twig litter	0.147	.001

There is some evidence that abundances of *G*. *pulex* and *G*. *d*. *celticus* were associated with different environmental conditions: the latter was related negatively to variation on axis 4 and positively to axis 1, whereas the former was related negatively to axis 1 and positively to axis 2. Similarly, abundance of *G*. *pulex* was related negatively to factor 1 but positively to factors 5 and 3, and abundance of *G*. *d*. *celticus* was related positively to variation in factor 3 and negatively to factors 4, 1, and 2.

### 3.4 Niche breadth/abundance relationships

**FIGURE 2:**
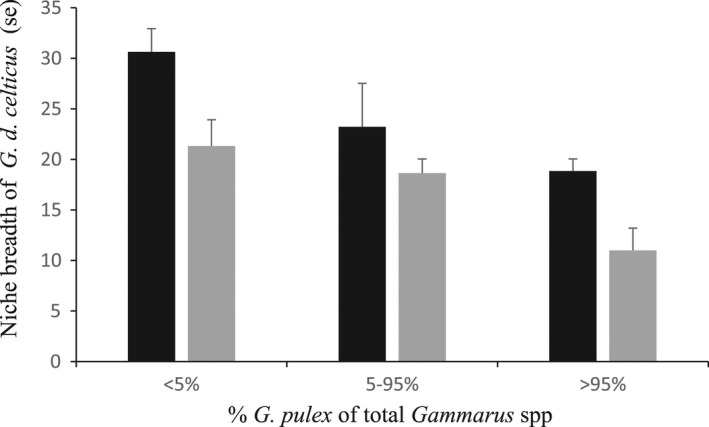
Mean (SE) niche breadth of *Gammarus duebeni celticus* at 20 sites categorized by presence of the invasive species *G*. *pulex*. Using %, all *Gammarus* spp. sites were placed in three categories: those with <5%, between 5 and 95%, and more than 95% *G*. *pulex* Niche breadth based on factorial analysis (FA) of physical and biotic environmental data (black) and axes from ordination (DCA) of invertebrate community data (gray columns)

Niche breadth of *G*. *pulex* when measured using community data and ordination (DCA) was related positively to intraspecific abundance but unaffected by the abundance of *G*. *d*. *celticus* (Table 4). Niche breadth of *G*. *d*. *celticus*, regardless of whether it was based on ordination (DCA) or environmental data (FA), was significantly, positively associated with intraspecific abundance and negatively with the abundance of *G*. *pulex* (Table 4). Niche breadths of *G*. *pulex* and *G*. *d*. *celticus* were positively correlated at the 15 sites where both species were present (*r_s_* = 0.73, *p* = .002 DCA; *r_s_* = 0.58 *n* 15, *p* = .049 FA). Analyses of variance, conducted on niche breadth measurements of *G*. *d*. *celticus* where sites were sorted into those with <5%, between 5 and 95%, and more than 95% *G*. *pulex*, indicated that niche breadth was greatest where the native species predominated, decreased where both species were present and was least where *G*. *d*. *celticus* occurred as a fraction of the overall gammarids present (Figure 2; overall niche breadth based on FA, *F* = 8.45, df = 2,17, *p* < .005; overall niche breadth based on DCA, *F* = 4.14, df = 2,17, *p* < .05). Niche breadth of *G*. *pulex* did not differ significantly between single species and mixed species sites. There was no overall difference between mean breadth of *G*. *pulex* (mean 18.13 SE 1.65 DCA; 24.36, SE 1.50, FA) and *G*. *d*. *celticus* (mean 17.84, SE 1.32 DCA; 24.35, SE 1.28, FA) across all sites using either measure of niche breadth.

**TABLE 4:** Spearman rank correlation of the relationship between numbers of *Gammarus pulex* and *G*. *duebeni celticus* and niche breadth at 25 sites. Overall niche breadth was measured as the sum over all axes (DCA) or factors (FA). One site had no gammarids

Abundance		
Niche breadth	*G. pulex*	*G. d. celticus*
DCA		
*G. pulex*	0.475*p* < .02	0.103
*G. d. celticus*	−0.196	0.583 *p* < .005
FA		
*G. pulex*	0.113	0.145
*G. d. celticus*	−0.406 *p* < .05	0.409 *p* < .05

## 4 DISCUSSION

The present study quantifies multidimensional niche breadth of native and invasive species along gradients using ordination of invertebrate community data (DCA) and factor analysis (FA) of environmental variables. We used these in unison to elucidate the effects of abundance of native and invasive species on niche breadth. Ordination and factor analyses, independently and together, suggest that a limited portion of community variation is determined by environment, notably: substrate heterogeneity and dominance, live vegetation and leaf litter, stream width and depth, grassy versus woody banks, and twig litter, grass vs woody cover, and width and depth. Environmental variation embraced no more than 15% variation in invertebrate samples with community data (DCA) having slightly less explanative power than measures of environmental variables (FA). However, niche breadth of the native *G*. *d*. *celticus* was related positively to its own abundance and negatively to abundance of *G*. *pulex*; the converse interspecific abundance effects on niche breadth of the invasive *G*. *pulex* were not evident in the analyses. This is consistent with the hypothesis that interspecific competition is occurring and that *G*. *d*. *celticus* is a weaker competitor in the field, being replaced by the competitively superior invader *G*. *pulex*. The process of replacement is rapid with a reduction in abundance of *G*. *d*. *celticus* in shared sites which had fewer *G*. *d*. *celticus* than *G*. *d*. *celticus* only species sites. *G*. *pulex*, on the other hand, increases in abundance rapidly when it appears in a stream, such that numbers in shared sites are similar to those where full replacement has already occurred. The replacement of *G*. *d*. *celticus* by *G*. *pulex* continued after the end of the present study (MacNeil & Briffa, 2009; MacNeil, Dick, et al., 2004; MacNeil, Prenter, et al., 2004). Positive relationships between niche breadth and conspecific population density (Faulks et al., 2015) have been reported, as have interspecific interactions involving related native species (Tarjuelo et al., 2017). The negative relationship between niche breadth of a native species and abundance of an invasive species has not been documented systematically in previous studies, although niche characteristics are known to be a factor in species invasions (Comte et al., 2017; Jackson et al., 2016; Pettitt‐Wade et al., 2015, 2018). Niche shifts and divergences have also been described in earlier studies of gammarid communities where two or more species coexist (for example, Herkül et al., 2016; Kley & Maier, 2005; Kotta et al., 2013). The present study suggests that changes in the behavior and distribution of the invaded native population, bringing about species replacement leading to a single species gammarid community, occur very soon after initial contact with an invasive species. This is in keeping with the rapidity of replacement of *G*. *d*. *celticus* by *G*. *pulex* in laboratory studies and simulations as well as rate of species replacement at sites between surveys (Dick et al., 1993, 1994; MacNeil & Briffa, 2009; MacNeil, Dick, et al., 2004; MacNeil, Prenter, et al., 2004). These changes are often difficult to detect, not least because the initial contact period is short and is followed by rapid change at the population level. Most species invasions are apparent only after invasive species are well established and already having obvious impact at the population and community level (Dickey et al., 2020; Simberloff et al., 2013).

*Gammarus duebeni celticus* is regarded as a widely distributed, generalist species with broader physiological tolerances than other brackish and freshwater *Gammarus* spp. (Gaston & Spicer, 2001). However, Prenter et al. (2004) have shown that *G*. *d*. *celticus* is less tolerant and more susceptible in precopula pair disruption than the invasive *G*. *pulex* in the presence of ammonia pollution, suggesting that environment is important in the species relationship and potential for coexistence. Further, Piscart et al. (2009) showed that the intraguild (IGP) interaction between *G*. *pulex* and *G*. *d*. *celticus* was mediated by water quality, while Piscart et al. (2011) concluded that native species of amphipods that coexist show interference competition, while intraguild predation is important in species replacement by invasive species. The river Lagan, as with many aquatic systems undergoing this *G*. *pulex*/*G*. *d*. *celticus* interaction (MacNeil et al., 2009), experienced a rapid species replacement of *G*. *d*. *celticus* by *G*. *pulex* associated with parasitism, cannibalism, and intraguild predation. Here, we add interspecific competition based on habitat structure as a contributing factor in gammarid species replacement (Dick & Elwood, 1992; Dick et al., 1993; Dunn & Dick, 1998; MacNeil et al., 2003). The present results suggest that the first sign of species replacement may be a gradual squeeze on niche parameters of the species being replaced as population growth of the invasive species starts to accelerate.

A “niche‐based approach to ecological risk assessment” has been proposed by Colas et al. (2014). This entails investigation of expansion or contraction of spatial and trophic niche breadth along disturbance gradients. Focusing on niche breadth with respect to conserving vulnerable species or controlling invasive species is also highlighted by Faulks et al. (2015) working on fish in Swedish lakes. They found a positive occupancy–abundance relationship related to wide isotopic (trophic) niche breadth. Similarly, Pettitt‐Wade et al. (2015) reported that “broad and plastic niche” enhances establishment of invasive fish species. This was supported by Comte et al. (2017) using stable isotope analysis to show that invasion by non‐native fish species was facilitated by plasticity in trophic ecology beyond that shown in their native ranges. In contrast, Jackson et al. (2016) demonstrated constriction of dietary niche in an invasive crab competing with a functionally similar native species. Pettitt‐Wade et al. (2018) also highlight the inconsistency in the link between niche breadth and invasion success focusing on differences between fish and aquatic invertebrates. Herkül et al. (2016) suggest that an invasive gammarid *G*. *tigrinus* has a narrower and more divergent realized niche than native gammarid species in coastal, northeastern areas of the Baltic Sea. Thus, it is important to consider the physical environment as well as the biological or ecological context of biological invasions in managing impacts of invasive species. While counterintuitive, niche breadths of *G*. *pulex* and *G*. *d*. *celticus* were positively correlated in shared sections of the river Lagan. This possibly reflects prevailing physical or trophic conditions. Niche breadth of the native *G*. *d*. *celticus* was affected negatively by increasing abundance of the invasive *G*. *pulex*. This might simply be a product of higher efficiency in some trophic role, for example, shredding, in the invading species (Truhlar et al., 2014). However, much more research will be required to establish general patterns in the relationship between niche breadth and abundance in invasive and native populations, and the environmental or species traits influencing departure from these norms.

We conclude that niche shifts may occur generally at an early stage in species replacements of native by an invasive species, as a result of competitive processes leading to the eventual demise of weaker competitors faced with novel, invasive species. This observation addresses the spatial or Hutchinsonian niche, but similar effects have been recorded with respect to the Eltonian or trophic niche despite contradictory results for aquatic invertebrates. Density dependence in niche parameters involving potentially competing species in general, and invasive species in particular, warrant closer scrutiny and may prove a valuable tool in managing vulnerable species as well as invasive and pest species.

## CONFLICT OF INTEREST

The authors declare that this research was conducted outwith commercial and/or financial concerns and is free of potential conflicts of interest.

## AUTHOR CONTRIBUTIONS

**W. Ian Montgomery:** Conceptualization (equal); Data curation (equal); Formal analysis (equal); Funding acquisition (equal); Investigation (equal); Methodology (equal); Writing – original draft (equal); Writing – review & editing (equal). **Bob W. Elwood:** Conceptualization (equal); Data curation (equal); Formal analysis (equal); Funding acquisition (equal); Project administration (equal); Writing – original draft (equal); Writing – review & editing (equal). **Jaimie T. R. Dick:** Conceptualization (equal); Data curation (equal); Formal analysis (equal); Funding acquisition (equal); Investigation (equal); Methodology (equal); Writing – original draft (equal); Writing – review & editing (equal).

## Supporting information

Table S1‐S3

## Data Availability

All data are available from the Dryad Digital Repository. https://doi.org/10.5061/dryad.j9kd51cdz.
