# Supplementary material for: Invader abundance and contraction of niche breadth during replacement of a native gammarid amphipod
Source: Ecol Evol. 2022 Mar 7;12(3):e8500. doi: 10.1002/ece3.8500 (PMC8928895; doi:10.1002/ece3.8500)
Supplement: Supplementary file 1 — Table S1‐S3 [file ECE3-12-e8500-s001.docx]

TABLE S1. Physical and biotic environmental variables measured.

| Environmental variable | Abbrev | Physical/  biological | Units | Description |
| --- | --- | --- | --- | --- |
| Water velocity | speed | physical | m/sec | flow rate of twig over measured distance |
| Corrected water velocity | corr speed | physical | m/sec | speed – mean flow rate during dry period (see Methods) |
| Maximum water depth | depth | physical | M | maximum depths of main channel |
| Corrected water depth | corr  depth | physical | M | depth - mean depth at site during dry period  (see Methods) |
| Maximum stream width | width | physical | M | maximum bank to bank width |
| Dominant substrate size | dom sub | physical | Ordinal | fine to coarse substrate on scale 1 – 5 corresponding to silt to rocks |
| Number of substrate types | no  sub | physical | numerical | total substrate types |
| Live aquatic vegetation | live  veg | biological | Ordinal | quantity attached moss and macrophytes on scale of 1 (absent) to 5 (abundant) |
| Marginal grass | grass | biological | Percent | 0-5m bankside percent grass cover |
| Overhanging grass | grass over | biological | Ordinal | Grass overhanging stream edge. Scale 1 (no overhang) to 5 (continuous overhang) |
| Marginal bush | bush | biological | Percent | 0-5m bankside percent bush cover |
| Overhanging bush | bush over | biological | Ordinal | Bush overhanging stream edge. Scale 1 (no overhang) to 5 (continuous overhang) |
| Marginal tree | tree | biological | Percent | 0-5m bankside percent tree cover |
| Overhanging tree | tree over | biological | Ordinal | Tree overhanging stream edge. Scale 1 (no overhang) to 5 (continuous overhang) |
| Leaf wet weight | leaf wt | biological | G | Wet weight of leaf retained in kick sample |
| Twig wet weight | twig wt | biological | G | Wet weight of woody material retained in kick sample |

TABLE S2 Between site variation in abundance of Gammarid spp. and invertebrates recovered from the River Lagan. Results (Anova F statistic) for 26 sites with 30 samples at each site.

| Order | Species/Family | F _25,754_ | P< |
| --- | --- | --- | --- |
| Amphipoda | *Gammarus pulex* | 105.03 | 0.0001 |
|  | *G.duebeni celticus* | 73.46 | 0.0001 |
| Isopoda | Asellidae | 99.95 | 0.0001 |
| Trichoptera | Glossosomatidae | 44.53 | 0.0001 |
|  | Hydropsychidae | 29.21 | 0.0001 |
|  | Leptoceridae | 8.43 | 0.0001 |
|  | Limnephilidae | 18.18 | 0.0001 |
|  | Polycentropidae | 6.79 | 0.0001 |
|  | Rhyaciphilidae | 8.57 | 0.0001 |
|  | Sericostomatidae | 6.80 | 0.0001 |
| Ephemeroptera | Baetidae | 35.54 | 0.0001 |
|  | Caenidae | 44.70 | 0.0001 |
|  | Heptageniidae | 53.10 | 0.0001 |
| Plecoptera | Capriidae | 3.76 | 0.0001 |
|  | Chloroperilidae | 30.91 | 0.0001 |
|  | Taeniopterypidae | 6.15 | 0.0001 |
| Diptera | Chironomidiae | 44.49 | 0.0001 |
|  | Empididae | 6.84 | 0.0001 |
|  | Muscidae | 10.25 | 0.0001 |
|  | Psychodidae | 2.96 | 0.0001 |
|  | Ptychopteridae | 1.00 | NS |
|  | Simulidae | 21.13 | 0.0001 |
|  | Syrphidae | 1.00 | NS |
|  | Tipulidae | 38.51 | 0.0001 |
|  | Trichoceridae | 1.42 | NS |
| Hemiptera | Corixidae | 5.32 | 0.0001 |
| Coleoptera | Curculionidae | 11.90 | 0.0001 |
|  | Dytiscidae | 4.13 | 0.0001 |
|  | Elminthidae | 49.63 | 0.0001 |
|  | Gyrinidae | 14.41 | 0.0001 |
|  | Haliphidae | 7.64 | 0.0001 |
|  | Helodidae | 1.00 | NS |
| Megaloptera | Sialidae | 1.93 | 0.01 |
| Odonata | Coenagriidae | 1.00 | NS |
| Acariformes | Hydrocarina | 8.92 | 0.0001 |
|  | Oribatei | 0.96 | NS |
| Pulmonata | Ancylidae | 32.98 | 0.0001 |
|  | Lymnaeidae | 58.42 | 0.0001 |
|  | Physidae | 9.83 | 0.0001 |
|  | Planorbiidae | 24.31 | 0.0001 |
| Prosobranchia | Hydrobiidae | 101.91 | 0.0001 |
|  | Valvatidae | 5.12 | 0.0001 |
| Cyrenodonta | Sphaeriidae | 29.21 | 0.0001 |
| Heptotaxida | Lumbricidae | 4.46 | 0.0001 |
| Rhynchobdellida | Glossiphoniidae | 15.75 | 0.0001 |
| Pharyngobdellida | Erpobdellidae | 29.64 | 0.0001 |
| Tricladida | Dendrocoelidae | 18.80 | 0.0001 |

TABLE S3 Between site variation in physical and biotic environmental variables on the river Lagan. Results (Anova F statistic) for 26 sites with 30 samples at each site.

| Environmental variable | F _25,754_ | P < |
| --- | --- | --- |
| Water speed | 22.1 | 0.0001 |
| Corrected speed | 4.6 | 0.0001 |
| Water depth | 55.9 | 0.0001 |
| Corrected depth | 35.0 | 0.0001 |
| Stream width | 215.9 | 0.0001 |
| Dominant maximum substrate | 23.5 | 0.0001 |
| Substrate heterogeneity | 9.2 | 0.0001 |
| Live aquatic vegetation | 16.9 | 0.0001 |
| Overhanging grass | 45 | 0.0001 |
| Overhanging bushes | 10.8 | 0.0001 |
| Overhanging trees | 23.3 | 0.0001 |
| Grass land use | 183.1 | 0.0001 |
| Bush land use | 11.5 | 0.0001 |
| Tree land use | 25.6 | 0.0001 |
| Weight of leaves | 6.4 | 0.0001 |
| Weight of twigs | 4.2 | 0.0001 |
